# Framework to Estimate Total Particulate Mass and Nicotine Delivered to E-cig Users from Natural Environment Monitoring Data

**DOI:** 10.1038/s41598-019-44983-w

**Published:** 2019-06-19

**Authors:** Edward C. Hensel, Nathan C. Eddingsaas, A. Gary DiFrancesco, Shehan Jayasekera, Sean O’Dea, Risa J. Robinson

**Affiliations:** 1 Department of Mechanical Engineering, Kate Gleason College of Engineering, Rochester Institute of Technology, Rochester, New York 14623 USA; 2 School of Chemistry and Materials Science, College of Science, Rochester Institute of Technology, Rochester, New York 14623 USA

**Keywords:** Predictive markers, Mechanical engineering

## Abstract

A framework describing the joint effect of user topography behavior and product characteristics of one exemplar device on the total particulate mass (TPM) and aerosol constituent yield delivered to a user is presented and validated against seven user-specific ‘playback’ emissions observations. A pen-style e-cig was used to collect emissions across puff flow rates and durations spanning the range observed in the natural environment. Emissions were analyzed with GC-MS and used to construct empirical correlations for TPM concentration and nicotine mass ratio. TPM concentration was demonstrated to depend upon both puff flow rate and duration, while nicotine mass ratio was not observed to be flow-dependent under the conditions presented. The empirical model for TPM and nicotine yield demonstrated agreement with experimental observations, with Pearson correlation coefficients of r = 0.79 and r = 0.86 respectively. The mass of TPM and nicotine delivered to the mouth of an e-cig user are dependent upon the puffing behavior of the user. Product-specific empirical models of emissions may be used in conjunction with participant-specific topography observations to accurately quantify the mass of TPM and nicotine delivered to a user.

## Introduction

**Figure 1:**
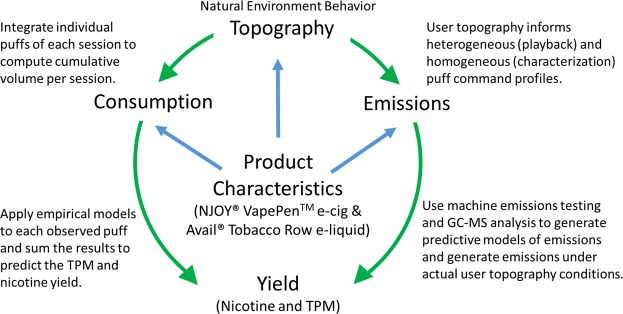
The yield of total particulate matter and nicotine delivered to an ENDS user’s mouth are dependent upon the user’s puff flow rate, puff duration, cumulative puffing volume and product characteristics of the ENDS and the e-liquid.

The purpose of this work is to develop an approach to predict on a per-person, per device, per consumable basis the total particulate mass and nicotine yield delivered to the mouth of an Electronic Nicotine Delivery System (ENDS) user, given knowledge of the tobacco product characteristics and the user’s topography and consumption behavior. The framework is informed by Fig. 1. Some previous reports have indicated that ENDS emissions are independent of flow rate^1,2^, while others have reported a dependence upon flow rate^3–5^, e-liquid solvent composition^6^, coil operating power, voltage or temperature^4,6–9^, device type^8,10^, or e-liquid flavor additives^7,9,11^. Reports have documented the presence of constituents in the aerosol which are present in the un-puffed e-liquid^7,11^, and also those which likely result from thermal decomposition processes^7,11^. One report of emissions generated from ‘playback’ of hookah topography profiles has demonstrated that aerosol emissions from water pipe are different for homogeneous (repeated puffs of constant duration and flow rate) and heterogeneous (puffs of individually varying duration and flow rate) puffing profiles^12^. Emissions from ‘playback’ natural environment topographies have not been previously reported for ENDS. There is a need to develop a quantifiable relationship between the joint effects of user behavior and product characteristics on the Harmful and Potentially Harmful Constituents (HPHC) delivered to a user’s mouth by ENDS. There is a further need to develop a framework by which emissions data reported by independent researchers may be compared to one another (to assess repeatability) and consolidated to develop a comprehensive understanding of the interplay between tobacco product characteristics, user behavior, and aerosol emissions.

## Approach

We define the cumulative yield, Y_AC_, of individual Aerosol Constituents (AC) of emissions delivered from an ENDS to the mouth of a user as the integral of the product of the time dependent mass ratio of the aerosol constituent, the Total Particulate Matter (TPM) concentration of the whole aerosol, and the user’s volumetric flow rate:

1 $${Y}_{{\rm{AC}}}\equiv {\int }_{{t}_{{\rm{initial}}}}^{{t}_{{\rm{final}}}}{f}_{{\rm{AC}}}(t){C}_{{\rm{TPM}}}(t)\dot{v}(t)dt$$

where the mass ratio of the constituent f_AC_(t) = m_AC_/m_TPM_ [mg/mg] and TPM Concentration C_TPM_(t) = m_TPM_/v [mg/mL] (mass per volume) vary with time as a user changes puffing patterns, tobacco product choices, and user-selectable device settings. The constituents in the ENDS aerosol may be present in the un-puffed e-liquid, or generated as decomposition products. We normalize all aerosol constituents (including vapor phase constituents, compounds originating in the e-liquid, and thermal decomposition products) by the mass of TPM emissions to facilitate separation of variables between the f_AC_ and C_TPM_ terms. We posit the TPM concentration and mass ratio of constituents may be expressed as linearly independent functions of Product Characteristics (PC) and User Behavior Characteristics (UBC). Numerous ENDS PC may affect the C_TPM_ including but not limited to the device operating power (reflected in coil wattage, amperage, or temperature), flow path geometry, coil design, and aspiration features. Additionally, the solvent composition of the e-liquid consumable (such as the PG/VG ratio which directly impacts the saturation temperature of the e-liquid) may impact C_TPM_. Additional consumable PC impacting f_AC_ may include nicotine concentration, flavor additives, viscosity, and pH. Furthermore, a variety of UBC may affect the C_TPM_ and/or f_AC_ including puff duration, d, flow rate, q, volume, v, and interval, i:

2 $${C}_{{\rm{TPM}}}={ {\mathcal F} }_{{\rm{TPM}}}(PC,UBC)$$

3 $${f}_{{\rm{AC}}}={ {\mathcal F} }_{{\rm{AC}}}(PC,UBC)$$

For the current study we limit variability in PC by selecting a single ENDS with no user-adjustable settings and a single e-liquid. We thus focus on the interaction between UBC and the flow path PC, reflected by the topography parameters q and d, and consider a single AC, nicotine, to illustrate the approach. Prior work^4^ demonstrated a power law relationship between C_TPM_ [mg/mL] and puff flow rate, q [mL/s]. Therefore, we propose the form of Eq. 4 to account for puff flow rate, q [mL/s], puff duration, d [s], and the product of those terms, which has physical significance as the puff volume v = q d [mL]. A transformation of variables enables a linear systems model describing the model-predicted TPM concentration, ${\hat{{\rm{C}}}}_{{\rm{TPM}}}$, of a single puff in terms of a set of empirical coefficients, b:

4 $$\mathrm{ln}({\hat{C}}_{{\rm{TPM}}})={b}_{1}+{b}_{2}\,\mathrm{ln}(q)+{b}_{3}(d)+{b}_{4}(\mathrm{ln}\,{(q)}^{2})+{b}_{5}\,\mathrm{ln}(d/1000)+{b}_{6}(q\cdot d)$$

The experimental observations of C_TPM_ can be computed as the ratio of the mass emissions captured on a filter pad per measured volume of aerosol passing through the pad. The coefficients in Eq. 4 may be estimated using ordinary least squares (OLS), weighted least squares (WLS), or other regression techniques. OLS regression is employed in the current work. Since the ENDS device chosen for the study does not have a user-selectable power setting, and the nicotine concentration of the e-liquid is held constant across all trials, we hypothesize a first order linear model, Eq. 5, for the model-predicted nicotine mass ratio, ${\hat{{\rm{f}}}}_{{\rm{NIC}}}$, as a function of puff flow rate, q.

5 $$\widehat{{f}_{{\rm{NIC}}}}={\beta }_{1}+{\beta }_{2}(q)$$

The regression coefficients, *β*, are also determined using OLS.

## Methods

### Aerosol generation, capture and analysis

The ENDS used for the current study was an NJOY vape pen e-cigarette with a top-fill (top-coil) e-liquid tank and no user adjustable settings such as power or coil temperature. The same model of ENDS was used for the emissions testing and natural environment monitoring of all results presented here. Similarly, the e-liquid used was AVAIL brand Tobacco Row (TR) having a manufacturer’s labeled nicotine concentration of 1.8% (measured using GC-MS to be 0.0140 ± 0.00014 [mg/mg] in the un-puffed e-liquid) and a measured solvent mixture ratio of 50:50 propylene glycol to glycerin. The nominal coil resistance of the vape pens used in the study ranged from 2.3 ± 0.3 (95% CI) ohms across nine coils prior-to and following the test series. The battery was recharged at the beginning of each test series, so device power was comparable between experimental conditions and repeated trials.

The PES-1 Programmable Emissions System was used to generate and collect aerosol emissions. The machine can be configured to collect particulate, vapor or liquid phase emissions from ENDS. A vacuum box can accommodate pressures as low as −25 [kPA], and hold sampling bags up to 5.0 [L] for mixed gas/liquid phase collection. The current study used a particulate phase collection mode with Cambridge style filter pads. The machine is driven by a vacuum pump (Model DOA-F704-AA, GAST) and flow is controlled by a proportional valve with response time of 10 milliseconds and range of 0–20 SLPM (KPIH-VP-20-156-25, Kelly Pneumatic Inc.). The system flow rate is measured using a precision gas flow meter (M-50SLPM-D-30PSIA/5M, Alicat Scientific, Inc.) third-party calibrated over its rated range of 20 SLPM. The emissions system is operated under direct digital control, enabling operation across a variety of flow conditions including the instantaneous puff profile, puff volume, flow rate, duration, and interval so that the machine can reproduce homogeneous (repeated) puff profiles or mimic heterogeneous (non-repeating) human puffing topographies for a range of behaviors.

The Total Particulate Matter (TPM) collected on filter pads following each emissions trial was determined gravimetrically using a Mettler AE240 Analytical Balance, with a protected weighing cell with a measurement accuracy of 0.0001 [g] and a repeatability of 0.1 [mg].

Nicotine mass ratio was determined using methods similar to those previously used in our lab^13^. In brief, pads were spiked with quinolone as an internal standard and submerged in methanol and broken up using orbital and wrist shaking followed by filtering through a 0.045 *μm* cellulose filter prior to GCMS analysis. In addition, calibration standards solutions of nicotine, that span the concentration range observed, and quinolone internal standard were prepared. The standard solutions were subjected to the same procedure as all samples, including introduction of a filter pad in each. Concentrations of nicotine were determined using a Shimadzu 2020 GCMS equipped with an AOC 20i autosampler. Triplicate runs of all samples and standards were run, 5 *μ*L samples and standards were sequentially injected into the GC and passed through a Restek Rxi-5 ms fused silica column (30 m × 0.25 mm I.D. × 0.25 *μ*m). Helium carrier gas was run through a split injector (50:1 split) at a temperature of 230C. The GC oven temperature was increased from 60C to 200C at 20C/min and then held for 3 minutes. The MS source and transfer line were kept at 180C and 280C, respectively, and the MS was run in the single-ion-monitoring mode (SIM) at m/z values of 133 (quantitation) and 162 (confirmation) for nicotine and 102 (quantitation) and 129 (confirmation) for quinolone. Peaks were integrated and the ratio of nicotine’s integration to its corresponding surrogate’s integration was used to establish calibration curves and determine nicotine concentrations in the samples. The mass of nicotine measured in each sample was divided by the mass of TPM measured on the corresponding filter pad to compute the measured nicotine mass ratio, f_NIC_.

### Method for quantifying the model coefficients

**Figure 2:**
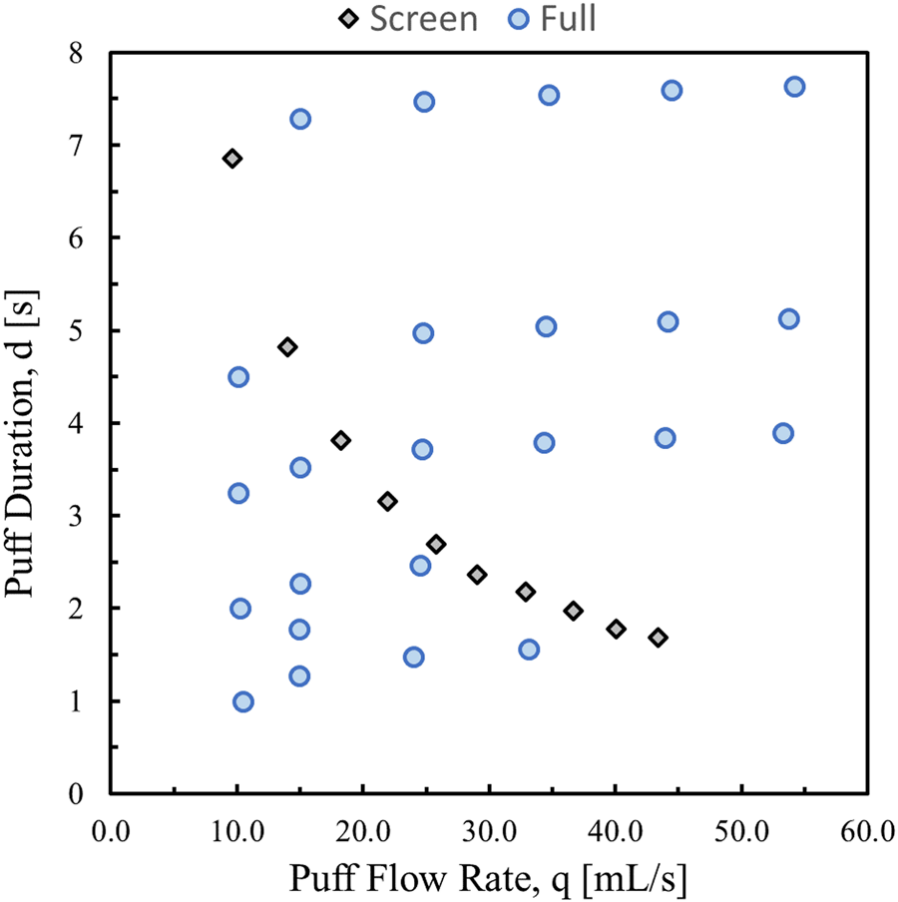
Design of experiments for emissions trials conducted as a function of measured puff flow rate, q, and puff duration, d, on an NJOY vape pen e-cigarette with AVAIL brand Tobacco Row e-liquid having a manufacturer’s labeled nicotine concentration of 1.8% and a solvent mixture ratio of 50:50 propylene glycol to glycerin. Ten screening and 24 additional flow conditions were investigated, with a mean session volume of approximately 740 [mL].

The empirical model coefficients for ${\hat{{\rm{C}}}}_{{\rm{TPM}}}$ (Eq. 4) and ${\hat{{\rm{f}}}}_{{\rm{NIC}}}$ (Eq. 5) were quantified by characterizing the ENDS and e-liquid over the range of puffing behaviors as observed in the natural environment^14–16^. Figure 2 illustrates the design of experiments used for the emissions testing experiment. The emissions testing began with a ‘screening’ experiment^4^ of 10 flow conditions wherein the puff duration and puff flow rate were varied inversely to maintain a nominally constant puff volume and cumulative session volume. The previous study^4^ presented screening results for cig-alike, vape-pen, box-mod and pod-style electronic cigarettes under varying conditions of coil location, operating power, and e-liquid flavors. The current work further investigates the joint effects of puff flow rate and puff duration on emissions for a single device, and thus controlling coil location, operating power and e-liquid. Using knowledge from the screening experiment, 24 additional ‘full’ flow conditions were tested wherein puff flow rate and duration were varied. Each flow condition was repeated for six trials, resulting in a total of 204 trials across 34 flow conditions. Each trial consisted of a fixed number of repeated puffs such that the average cumulative aerosol volume generated per trial was approximately 740 [mL], ranging from 522 [mL] to 1000 [mL]. All results are presented in terms of observed actual flow conditions as opposed to the nominal programmed command conditions. The TPM mass was measured and divided by the cumulative observed aerosol volume associated with each trial to determine the value of C_TPM_ for each trial at each flow condition. The resulting observations of C_TPM_ as a function of q and d are used in conjunction with an ordinary linear least squares algorithm to estimate the coefficients ${b}_{1}\ldots {b}_{6}$ appearing in Eq. 4. The filter pad from selected ‘screening’ trials were analyzed using GC-MS to determine the mass ratio of nicotine to TPM in conjunction with a linear least squares algorithm to estimate the coefficients ${\beta }_{1}$ and ${\beta }_{2}$ appearing in Eq. 5.

### Method for validating the model

The model was validated by comparing predictions for ${\hat{{\rm{C}}}}_{{\rm{TPM}}}$ (Eq. 4) and ${\hat{{\rm{f}}}}_{{\rm{NIC}}}$ (Eq. 5) to experimental emissions observations of C_TPM_ and f_NIC_ generated by mimicking the natural environment puffing sessions of seven participants. The study was reviewed and approved by the Rochester Institute of Technology (RIT) Human Subjects Research Office Institutional Review Board (IRB) and the RTI International IRB, in compliance with relevant guidelines and regulations. Informed consent was obtained from all subjects and data for model validation were exported in a de-identified manner.

One natural environment puffing session ‘playback’ data set was selected for each of the seven participants in a previously reported two week switching study^14^ who elected to use e-liquid with a nicotine concentration of 1.8%. Each session listed in Table 1 was selected as representative of the topography behavior exhibited by the participant during six full days of ENDS use with TR e-liquid.

**Table 1:** Exemplar ‘playback’ puffing profiles informed by seven participants from a previous two week natural environment switching study used to validate the framework.

Ppt	Profile ID	Time [MM-DD hh:mm]	d [s]	$\bar{{\bf{q}}}$ [mL/s]	Volume [mL]
OS3-01	*α*	07–04 14:32	1.2	50.6	1007.0
OS3-06	*α*	08–11 21:44	1.2	18.4	830.0
OS3-10	*α*	09–06 09:06	3.0	16.1	503.0
OS3-12	*α*	09–08 12:36	1.2	30.8	1004.0
OS3-14	*α*	09–15 22:51	1.2	16.7	748.0
OS3-26	*α*	11–12 03:10	2.3	30.4	605.0
OS3-28	*α*	11–20 19:25	4.4	62.6	360.0

Each participant used an NJOY vape pen e-cigarette with AVAIL brand Tobacco Row e-liquid having a labeled nicotine concentration of 1.8% and a measured solvent mixture ratio of 50:50 propylene glycol to glycerin. The ‘Time’ column indicates the date and time the natural environment observation data was collected from each participant.

**Figure 3:**
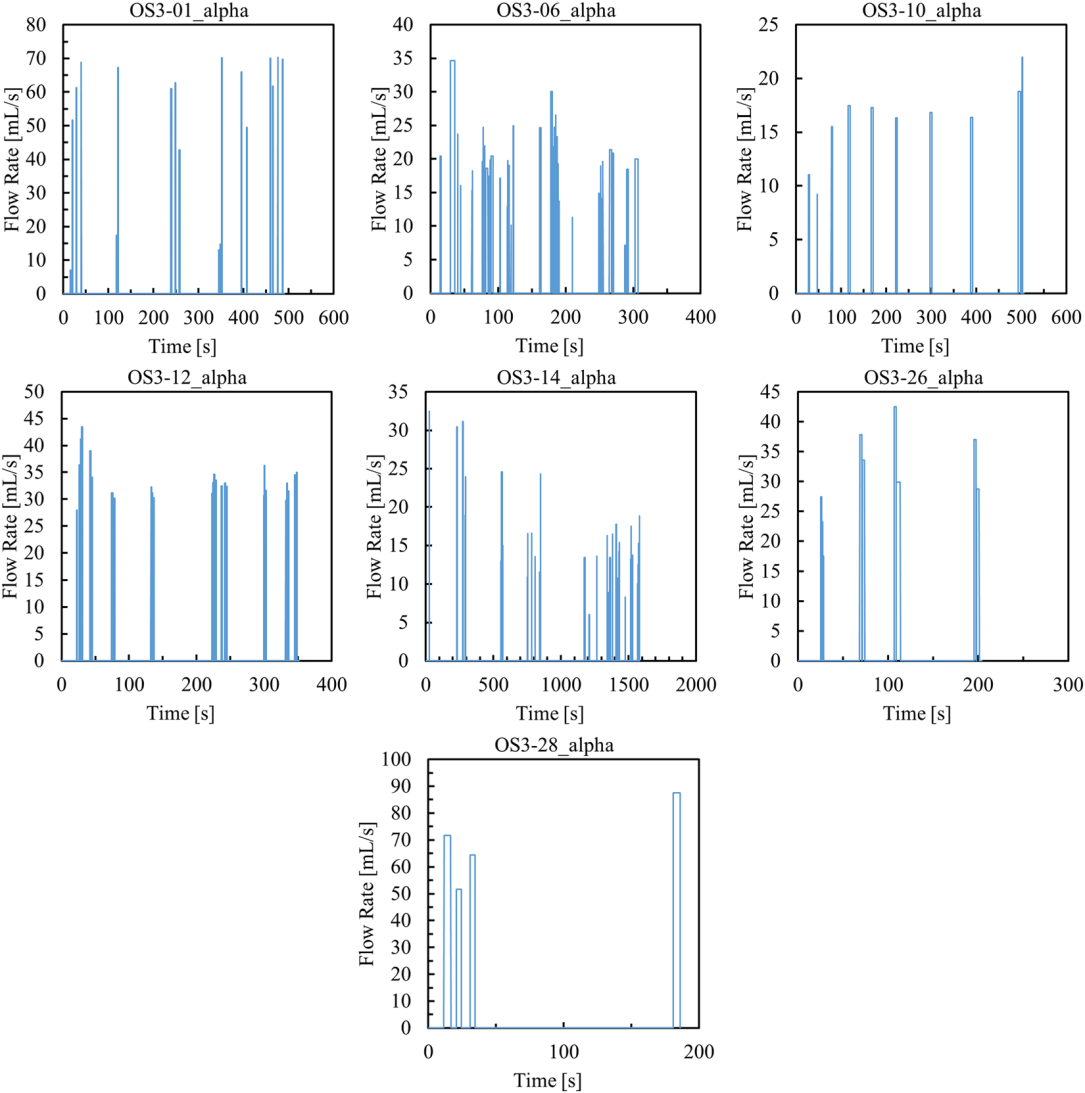
Exemplar natural environment topography ‘playback’ profiles for participants 01, 06, 10, 12, 14, 26, and 28 illustrating discrete puffs over a single ENDS use session for each participant.

Figure 3 illustrates exemplar puffing session data collected for seven participants in the natural environment during a recent two-week switching study. These observations were used to generate ‘playback’ command profiles to generate emissions and estimate the participant-specific yields of TPM and NIC.

Validation of the model consisted of the following sequence of operations.

1. Seven sessions representative of natural environment topography data (Table 1 and Fig. 3) were used to generate ‘playback’ puffing profiles as input to the PES-1 system.
2. The emissions system was operated with the subject ENDS device and e-liquid to mimic the behavior of each study participant. Each ‘playback’ puffing profile was repeated for six trials, with the device battery regularly recharged and the e-liquid tank confirmed to be greater than 1/2 full at the beginning of each trial.
3. The filter pads were measured after each of the 42 trials to determine the experimentally observed TPM yield, Y_TPM_.
4. Each filter pad was analyzed using GC-MS to determine the experimentally observed nicotine yield, Y_NIC_.
5. Apply the empirical model for ${\hat{{\rm{C}}}}_{{\rm{TPM}}}$ (Eq. 4) to each ‘playback’ puff, based on the actual q and d measured in the PES-1, and sum the individual puff results to estimate the TPM yield for each ‘playback’ session, wherein N_puff_ is the number of discrete puffs during each session:
  6 $${\hat{Y}}_{{\rm{TPM}}}=\sum _{n=1}^{{N}_{{\rm{puff}}}}{\hat{C}}_{TPM,n}({q}_{{\rm{puff}},n},{d}_{{\rm{puff}},n}){V}_{{\rm{puff}},n}$$
6. Apply the empirical model for ${\hat{{\rm{C}}}}_{{\rm{TPM}}}$ (Eq. 4) and ${\hat{{\rm{f}}}}_{{\rm{NIC}}}$ (Eq. 5) to each puff, and once again sum the results over all puffs to predict the nicotine yield, ${\hat{{\rm{Y}}}}_{{\rm{NIC}}}$:
  7 $${\hat{Y}}_{{\rm{NIC}}}=\sum _{n=1}^{{N}_{{\rm{puff}}}}{\hat{f}}_{NIC,n}({q}_{{\rm{puff}},n}){\hat{C}}_{TPM,n}({q}_{{\rm{puff}},n},{d}_{{\rm{puff}},n}){V}_{{\rm{puff}},n}$$
7. Compare predicted emissions yield to playback emissions yield (${\hat{{\rm{Y}}}}_{{\rm{TPM}}}$ vs Y_TPM_ and ${\hat{{\rm{Y}}}}_{{\rm{NIC}}}$ vs Y_NIC_) for the six repeated trials of seven selected validation sessions.
8. Report the slope, m, Pearson’s correlation coefficient, r, and coefficient of determination, R^2^, for model predictions vs experimental observations for TPM and nicotine yield. As each of these quantities approach unity we would reproduce the ideal 1:1 line and have perfect agreement between the empirical model and experimental data.

## Results

### Empirical coefficients in the framework

**Figure 4:**
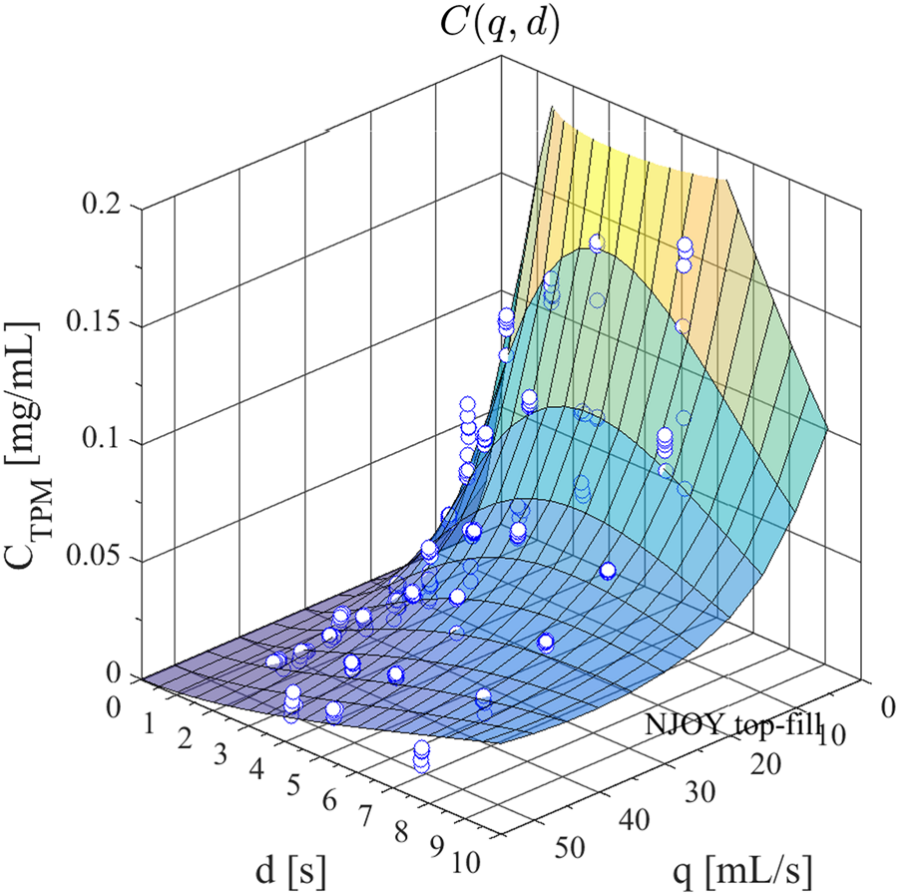
Total particulate matter concentration of emissions generated from NJOY vape pen e-cigarette with AVAIL brand Tobacco Row e-liquid having a labeled nicotine concentration of 1.8% and a measured solvent mixture ratio of 50:50 propylene glycol to glycerin as a function of observed puff flow rate and duration.

Figure 4 shows the results of the emissions study conducted using NJOY e-cig operated across a wide range of puff flow rates and puff durations, using Avail brand Tobacco Row flavored e-liquid with a manufacturer’s labeled nicotine concentration of 1.8%. Each trial is represented by a sphere, and the shaded surface illustrates the empirical model used to describe the C_TPM_ as a function of puff flow rate, q, and duration, d.

The linear regression coefficients in the empirical model for ${\hat{{\rm{C}}}}_{{\rm{TPM}}}$ (Eq. 4) are presented in Table 2. The ordinary coefficient of determination for the model is ${R}^{2}\approx 0.905$ and the adjusted value is ${R}_{adj}^{2}\approx 0.903$ with a root mean square error of 0.012 [mg/mL].

**Table 2:** Empirical model regression coefficients for ${\hat{{\rm{C}}}}_{{\rm{TPM}}}$.

Coefficient	Estimate	Std. Error	tStat	pValue
b1	9.513425	1.332116	7.141590	0.000000
b2	1.312385	0.594033	2.209278	0.028302
b3	−0.448513	0.051740	−8.668628	0.000000
b4	−0.581447	0.101911	−5.705444	0.000000
b5	1.775869	0.149039	11.915489	0.000000
b6	0.007221	0.000775	9.321067	0.000000

**Figure 5:**
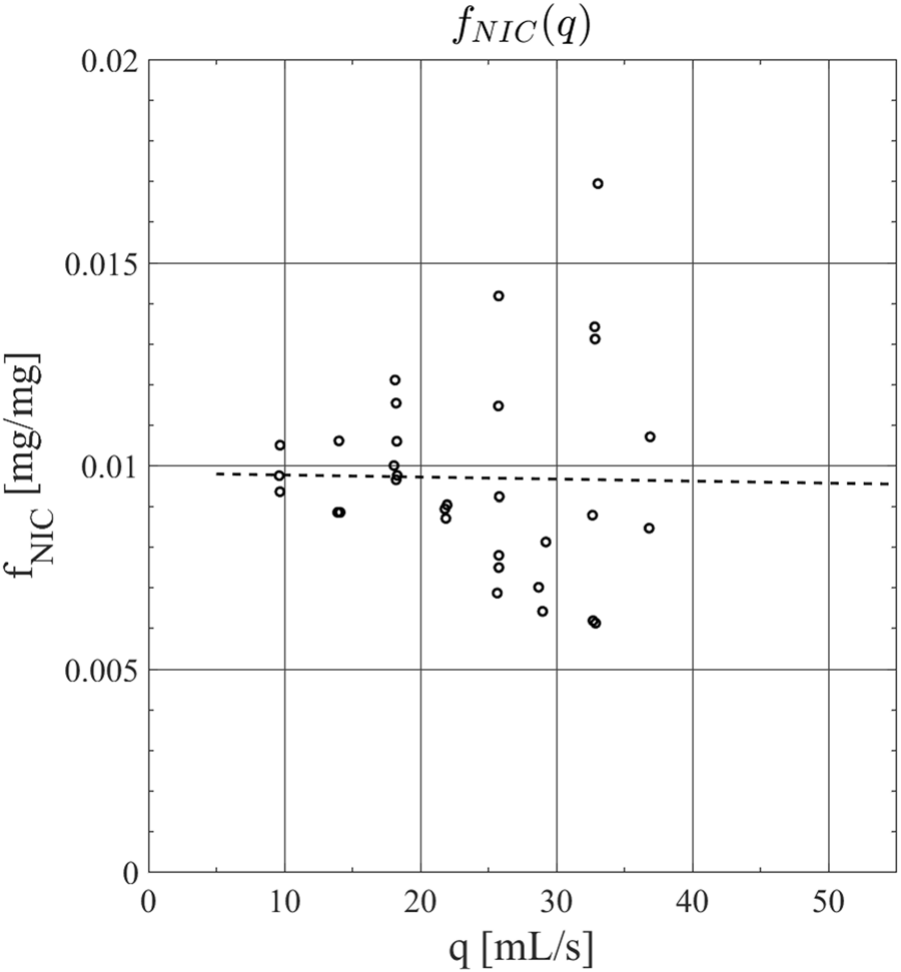
Mass ratio of nicotine emissions generated from an NJOY vap pen e-cigarette with AVAIL brand Tobacco Row e-liquid having a labeled nicotine concentration of 1.8% and measured solvent mixture ratio of 50:50 propylene glycol to glycerin as a function of observed puff flow rate.

Figure 5 shows the results of the GC-MS analysis for nicotine mass ratio present in the TPM emissions samples. Also shown is the empirical curve fit for the nicotine mass ratio as a function of puff flow rate. The linear least squares regression coefficients in the empirical model for ${\hat{{\rm{f}}}}_{{\rm{NIC}}}$ (Eq. 5) are shown in Table 3. The coefficient of determination for the model is R^2^ = 0.0002, reflecting the significant scatter in the observations, with a root mean square error of 0.0025 [mg/mg].

**Table 3:** Empirical model regression coefficients for ${\hat{{\rm{f}}}}_{{\rm{NIC}}}$.

Coefficient	Estimate	Std. Error	tStat	pValue
*β*_1_	0.009809	0.001369	7.167247	0.000000
*β*_2_	−0.000005	0.000055	−0.084850	0.028302

### Validation results

**Figure 6:**
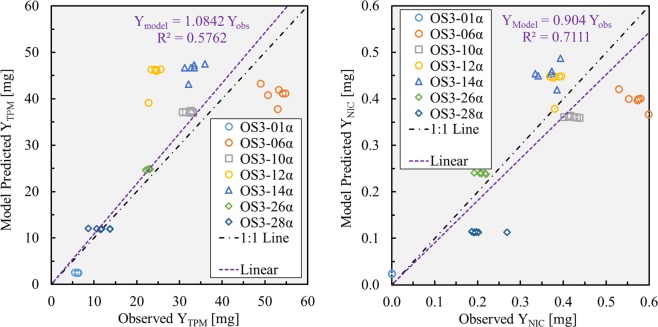
Experimental validation of total particulate matter yield (left) and nicotine yield (right) measured from lab playback machine puffing sessions compared to empirical model predictions.

Figure 6 illustrates the correlation of the empirical model estimates of ${\hat{{\rm{Y}}}}_{{\rm{TPM}}}$ and ${\hat{{\rm{Y}}}}_{{\rm{NIC}}}$ versus machine puffed playback measurements for six repeated trials of seven exemplar playback sessions. The slope for ${\hat{{\rm{Y}}}}_{{\rm{TPM}}}$ is m = 1.08, R^2^ = 0.58, and Pearson’s correlation coefficient of r = 0.79. The slope for ${\hat{{\rm{Y}}}}_{{\rm{NIC}}}$ is m = 0.904, R^2^ = 0.71 and a Pearson’s correlation coefficient of r = 0.86. The scale of the two validation figures varies by a factor of 100 due to the circumstance that ${\hat{{\rm{f}}}}_{{\rm{NIC}}}$ = 0.01 and is independent of flow rate for the e-cigarette and e-liquid tested here. The difference in the r value reflects experimental differences in the observations of Y_TPM_ and Y_NIC_.

## Discussion

Natural environment observations of user topography behavior have been used to create ‘playback’ command profiles and demonstrate estimates of user-specific exposure to harmful and potentially harmful constituents. The mass of particulate matter and nicotine delivered to the mouth of an electronic cigarette user are dependent upon the puffing topography behavior of the user. The total particulate matter concentration and nicotine mass ratio may be determined as a function of product characteristics and user behavior characteristics and used to create a predictive emissions model specific to each tobacco product. The predictive emissions model may be used in conjunction with user-specific topography observations to estimate the mass of particulate matter and aerosol constituents delivered to the mouth of an individual user. The proposed framework may be used to assess the effect of e-liquid and e-cigarette product characteristics on emissions. The proposed framework may be used to examine the relationship between product characteristics, user behavior characteristics, and user-specific exposure to whole aerosol mass and individual constituents there of.

A framework was introduced and validated. Total Particulate Matter (TPM) emissions were collected from a machine puffing system across the range of puff duration and puff flow rate typical of observed natural environment behavior. The TPM concentration was demonstrated to be a function of both puff flow rate, q, and puff duration, d. The nicotine mass ratio was determined across the range of puff duration and flow rate and found to be relatively independent of flow conditions for the products studied. The emissions data was used to create empirical models for ${\hat{{\rm{C}}}}_{{\rm{TPM}}}$ and ${\hat{{\rm{f}}}}_{{\rm{NIC}}}$. The model predictions of ${\hat{{\rm{Y}}}}_{{\rm{TPM}}}$ and ${\hat{{\rm{Y}}}}_{{\rm{NIC}}}$ yield were validated against experimental observations of Y_TPM_ and Y_NIC_ representative of natural environment behavior of ENDS users. The framework was demonstrated to be predict the yield of machine ‘playback’ emissions profiles with Pearson’s correlation coefficients of r = 0.79 (TPM) and r = 0.86 (NIC).

The framework provides a context to enhance the understanding of the functional relationship between topography and consumption behavior and product characteristics of ENDS and e-liquids to estimate the total particulate and HPHC constituent yield delivered to a user. The framework defines the constituent mass ratio and TPM concentration as a means of separating the influence of ENDS flow path and power characteristics from the composition of e-liquids, offering the potential for more robust data sharing between research groups and enabling data re-use. The ability to quantify the yield of TPM and HPHC delivered to an ENDS user enables the investigation of a causal relationship between the time-history product use and observed bio-markers of exposure (indicators of uptake). The approach may lead to the ability to predict a-priori the effects of changes in product characteristics on resulting emissions, yield, and uptake. It is recommended that TPM emissions test results be reported as the mass of TPM per volume of whole aerosol. It is recommended that HPHC emissions test results be reported as the ratio of mass of the HPHC per mass of TPM. The actual flow conditions, device parameters and e-liquid composition should be reported with all emissions and yield data.

The data and analysis presented in this manuscript are in terms of the “puffed aerosol” collected under various topography (puff flow rate and duration) conditions. The study employed e-liquids directly from manufacturer’s retail bottles, drawn from the same production lots as the e-liquids provided to participants in the preceding human subject study^14^. The emissions test results (Figs 4 and 5) and the experimental validation results (Fig. 6) were all conducted by refilling the vape pen tank from manufacturer’s packaging, reflecting the actual usage of the previous study participants.

While the approach is generally applicable to a wide variety of ENDS and e-liquids, the numerical results are not. The coefficients computed for the empirical functions describing ${\hat{{\rm{C}}}}_{{\rm{TPM}}}$(q, d) and ${\hat{{\rm{f}}}}_{{\rm{NIC}}}$(q) are specific to emissions collected with an NJOY e-cigarette filled with AVAIL brand Tobacco Row e-liquid having a labeled nicotine concentration of 1.8% and a solvent mixture ratio of 50:50 propylene glycol to glycerin. The functional form of ${\hat{{\rm{C}}}}_{{\rm{TPM}}}$(q, d) and ${\hat{{\rm{f}}}}_{{\rm{NIC}}}$(q) are anticipated to be device and consumable dependent, particularly in the case of ENDS with adjustable power and/or flow path settings. The single model of vape pen presented in this study has no user-selectable power adjustment options, so any variation in device power which may have been induced by variations in coil resistance between test articles and over time are reflected in the repeated trial variability present in the reported experimental data. A full investigation of the electronic principles of operation and power management of electronic cigarettes is beyond scope of the current work and is deferred to a future study.
